# Mobile Phone Addiction and Academic Procrastination in Adolescents: The Serial Mediating Roles of Self‐Regulation and Psychological Resilience and the Moderating Role of the Parent‐Child Relationship

**DOI:** 10.1002/brb3.71169

**Published:** 2025-12-31

**Authors:** Yang Liu, Yan Lin, Shaokun Zhao, Fan Wang, Qingying Yuan, Yongsheng Tong

**Affiliations:** ^1^ Peking University Huilongguan Clinical Medicine School Beijing Huilongguan Hospital Beijing China; ^2^ Jinzhou Medical College Jinzhou Liaoning China

**Keywords:** academic procrastination, mobile phone addiction, parent‐child relationship, psychological resilience, self‐regulation

## Abstract

**Background:**

Mobile phone addiction and academic procrastination are two common behavioral challenges among adolescents. While prior research has documented their association, the underlying mechanisms—particularly the mediating roles of self‐regulation and psychological resilience and the moderating role of the parent‐child relationship —remain insufficiently examined.

**Methods:**

This study involved 966 adolescents who completed the Mobile Phone Addiction Scale, Self‐Regulation Scale, Brief Resilience Scale, Parent‐Child Relationship Scale, and Academic Procrastination Scale–Short Form. Data were analyzed using partial least squares structural equation modeling (PLS‐SEM).

**Results:**

The results of the research showed that (1) mobile phone addiction had a significant positive effect on academic procrastination; (2) self‐regulation mediated between mobile phone addiction and academic procrastination; (3) psychological resilience mediated between mobile phone addiction and academic procrastination; (4) self‐regulation and psychological resilience served as serial mediators between mobile phone addiction and academic procrastination and (5) parent‐child relationship significantly moderated the association of mobile phone addiction on academic procrastination.

**Conclusion:**

This study describes the further relationships among mobile phone addiction, academic procrastination, and related factors in adolescents. The observed patterns suggest that approaches to adolescent well‐being may benefit from integrated frameworks considering individual, familial, and educational dimensions—with a particular focus on the role of self‐regulation, psychological resilience, and parent‐child relationship.

## Graphical abstract

Based on Social Cognitive Theory (SCT), this study found that self‐regulation and psychological resilience functioned as serial mediators between mobile phone addiction and academic procrastination. Meanwhile, parent‐child relationship significantly moderated the effect of mobile phone addiction on academic procrastination.

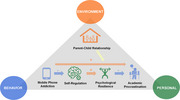

## 1 Introduction

Academic procrastination is typically defined as the behavior of individuals who are aware of the importance and urgency of an academic task but delay completing it due to lack of motivation, emotional distress, or task difficulty (Olleras et al. 2022). Procrastination is a widespread phenomenon in academic settings, adversely affecting academic performance (García‐Ros et al. 2023; Rahimi et al. 2023). In the long term, it also increases an individual's emotional burden and even creates a vicious cycle (Gadosey et al. 2024), further hindering the formation of learning habits and personal development of adolescents. Given the negative consequences of procrastination, identifying risk factors that may contribute to this issue is beneficial. In the digital age, mobile phone addiction has gradually become a key risk factor affecting adolescents’ academic performance. Excessive mobile phone use not only occupies a large amount of study time but is also closely linked to academic procrastination. Under the irreversible tide of digitalization, the synergistic effect between excessive mobile phone use and academic procrastination is likely to become increasingly pronounced in the coming years. Therefore, to advance understanding of mobile phone addiction and academic procrastination while informing the development of targeted prevention and intervention strategies, identifying the associative patterns and risk factors linking these two issues among adolescents is essential.

Although the association between mobile phone addiction and academic procrastination has gradually been noticed by scholars, the underlying psychological mechanisms remain poorly understood. Previous studies on academic procrastination has typically focused on isolated variables such as specific personality traits (e.g., the Big Five traits) (Hidalgo‐Fuentes et al., 2024) or cognitive factors (e.g., fear of failure) (Balkıs and Duru 2022), overlooking the complex interplay between personal psychological factors, behaviors, and environmental contexts. Bandura's Social Cognitive Theory (SCT) offers a valuable perspective to address this limitation. Its core principle of triadic reciprocity emphasizes dynamic interactions among personal, behavioral, and environmental factors (Bandura 2001). Guided by this framework, the present study investigates whether mobile phone addiction (behavior) predicts academic procrastination (behavior) through the mediating roles of self‐regulation (personal factors) and psychological resilience (personal factors) and whether the parent‐child relationship (environmental factor) moderates this pathway. By applying SCT to this multifaceted behavioral issue, this research aims to provide a more integrated understanding of these phenomena and extend the theory's application in contemporary digital contexts.

Previous studies have indicated that mobile phone addiction is associated with academic procrastination. Meta‐analytic findings confirm a moderate positive correlation between these constructs (Zhou et al. 2024). Empirical studies further demonstrate that heightened engagement with digital media can result in task postponement and duty evasion (Gökalp et al. 2023; Meier et al. 2016). Individuals may use smartphones as a means to satisfy unmet desires or escape negative emotions and external pressures, ultimately leading to procrastination on academic tasks (George et al. 2023). Grounded in SCT, this study aims to examine the relationship between the two within an integrated framework and elucidate its underlying mechanisms. Therefore, this study hypothesizes:

**Hypothesis 1**. *Mobile phone addiction would positively and significantly predict adolescent academic procrastination*.

Self‐regulation is defined as the ability of an individual to actively regulate his or her emotions, thoughts, and behaviors to adapt to the environment or achieve a goal (Wagner and Heatherton 2015). Within the current SCT framework, self‐regulation as a “personal factor” may play a significant role in the relationship between mobile phone addiction and academic procrastination among adolescents. Research indicates that excessive mobile phone use depletes self‐regulatory resources. According to flow theory, the constant stimulation and immediate rewards of smartphones can foster a state of immersion that weakens cognitive control and makes disengagement difficult (Jo and Baek 2023). This process can also activate adolescents’ socio‐emotional systems, making them more susceptible to emotional impulses (Kazanjian 2023), further undermining self‐discipline. In turn, low self‐regulation ability is associated with various negative psychological issues and poorer academic performance, including anxiety, depression, and academic procrastination (Kökönyei et al. 2024; Rad et al. 2025; Young et al. 2019). Low self‐regulation ability is a known risk factor for academic procrastination (Mohammadi Bytamar et al. 2020), as low self‐regulators may tend to focus more on their emotions and emotional responses rather than adopting a task‐oriented style, which results in postponing the aversive tasks (Steel 2007). Therefore, it is hypothesized that mobile phone addiction weakens self‐regulation, which subsequently increases academic procrastination. However, the mediating role of self‐regulation in this specific relationship remains unexplored. Thus, we propose:

**Hypothesis 2**. *Self‐regulation would demonstrate a significant mediating role in the association between mobile phone addiction and academic procrastination*.

Psychological resilience refers to an individual's adaptive ability to effectively regulate their emotions and behaviors when facing stress, challenges, and adversity (Troy et al. 2023), and it represents another key personal factor within our social cognitive framework. We propose that it may also serve as a crucial link between mobile phone addiction and academic procrastination. Generally speaking, resilient adolescents can effectively deal with academic and personal challenges while maintaining emotional balance. However, excessive mobile phone use may hinder the development of psychological resilience in adolescents (Quynh Ho et al. 2023). Chronic immersion in the digital world may reduce adolescents’ interaction with real‐world stressors, preventing adolescents from developing effective coping strategies for real‐life challenges, thereby diminishing their resilience over time (Brand et al. 2019; Domoff et al. 2020). In contrast, students’ psychological resilience is positively correlated with their commitment to academic tasks (Sakız and Aftab 2019). Lack of psychological resilience often increases the occurrence of negative emotions and avoidance behaviors under academic pressure, heightening the likelihood of academic procrastination (Skinner et al. 2020). Thus, a pathway is proposed whereby mobile phone addiction weakens psychological resilience, which in turn increases academic procrastination. However, to our knowledge, current research examining such associations among adolescent populations remains scarce. Based on this, the study hypothesized the following:

**Hypothesis 3**. *Psychological resilience would play an important mediating role in the association between mobile phone addiction and academic procrastination*.

In addition, self‐regulation is closely related to psychological resilience (Dias and Cadime 2017). Currently, psychological resilience is regarded as a non‐static trait underpinned by dynamic psychological processes, with self‐regulation potentially playing a crucial role in this process (Supervía et al. 2022). Under pressure, each successful use of self‐regulation strategies to overcome challenges strengthens an individual's sense of efficacy, which subsequently strengthens broader psychological resilience (Dahl et al. 2020). Consequently, mobile phone addiction may undermine psychological resilience by impairing self‐regulation, a pathway that ultimately leads to increased academic procrastination. While this sequential pathway is theoretically plausible, it has not been empirically tested in adolescent populations. Therefore, the following research hypothesis is proposed:

**Hypothesis 4**. *Self‐regulation and psychological resilience would serve as serial mediators between mobile phone addiction and academic procrastination*.

According to SCT, environmental factors are also crucial in shaping behavior, in addition to personal factors. The parent‐child relationship, as a primary proximal environment, may therefore moderate the association between mobile phone addiction and academic procrastination. Parent‐child relationship is defined as the interactive patterns and emotional connection between parents and children (Ali et al. 2021). From the SCT perspective, the parent‐child relationship represents an environmental determinant that may shape behavioral regulation through emotional modeling and reinforcement processes (Butler et al. 2022). High‐quality parent‐child relationship, may serve as a protective buffer against maladaptive behaviors. Based on this, exploring the moderating role of parent‐child relationship in adolescents’ mobile phone addiction and academic procrastination is of distinct meaning. And we hypothesized the following:

**Hypothesis 5**. *Parent‐child relationship would moderate the correlation between mobile phone addiction and academic procrastination*.

**FIGURE 1:**
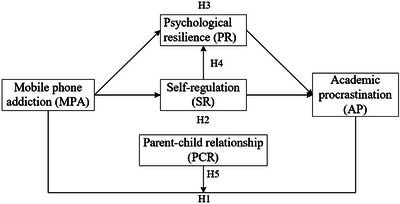
Hypothetical model.

In summary, while the associations between mobile phone addiction, self‐regulation, psychological resilience, and academic procrastination are recognized individually, their integration within a unified theoretical framework is lacking. To our knowledge, no study has simultaneously examined the multiple mediating roles of self‐regulation and resilience, along with the moderating effect of the parent‐child relationship. The present study addresses this gap by proposing and testing an integrated model grounded in SCT. This conceptual model is shown in **Figure** **1**.

## 2 Materials and Methods

### 2.1 Participants

This cross‐sectional study was conducted among junior high and high school adolescents in various schools across several provinces in China during February and March 2025. This study adopted a collaborative approach with schoolteachers, distributing questionnaire posters specifically within target parent/class WeChat groups. Questionnaires were distributed through an online survey platform (named “Sojump”) in a convenience sampling format. Participants were explicitly informed that their responses would be treated as fully anonymous, and the data would be used solely for academic research purposes with no access granted to the school or teachers. Before the survey, informed consent was obtained from students and their parents/guardians. To ensure questionnaire quality, a quality check item (“To ensure response quality of the questionnaire, please select ‘2’ for this question, which has five answer choices from ‘1’ to ‘5’.”) was added at the middle‐to‐latter part of the survey to reduce careless responding. A total of 1000 questionnaires were collected, and 34 questionnaires were excluded (incorrect responses to quality control questions). Thus, a total of 966 participants were analyzed in this study, including 190 male (19.7%) and 776 female (80.3%) students. All scales were completed by the adolescents themselves. The mean grade level was 3.91 (SD = 1.20; Median = 4). Strict ethical approval requirements are adhered to during the data collection process to ensure the confidentiality of their personal information.

### 2.2 Measures

#### 2.2.1 Mobile Phone Addiction Scale

In the current study, a revised version of the 11‐item Internet Addiction Scale was used to measure students’ mobile phone addiction (Hong et al. 2012; Young 1998). Items were rated on a 6‐point Likert scale ranging from 1 (incompletely agreed) to 6 (completely agreed). Higher scores reflected a higher degree of one's mobile phone addiction. In this study, the Cronbach's α coefficient was 0.942.

#### 2.2.2 Self‐Regulation Scale

The items of the scale were designed to reflect dispositional attention control and regulation. Each item is rated on a 4‐point scale ranging from 1 (not at all true) to 4 (completely true), and responses are summed into a total score, with higher scores indicating greater ability to self‐regulate (Diehl et al. 2006). In this study, Cronbach's α coefficient for self‐regulation was 0.915.

#### 2.2.3 Brief Resilience Scale

The 6‐item Brief Resilience Scale was used to assess the psychological resilience of individuals. Items were rated on a 5‐point Likert scale (1 = strongly disagree, 5 = strongly agree). A higher score is indicative of an elevated level of psychological resilience (Smith et al. 2008). The Cronbach's α of the scale was 0.896 in this study.

#### 2.2.4 Parent‐Child Relationship Scale

The Parent‐Child Relationship Scale consists of 8 items using 5‐point Likert questions (from “1 = never” to “5 = several times a day”) to assess the frequency of conflicts between participants and their parents over the past 6 months in the areas of family relationships, academics, housework, friendships, spending, diet and lifestyle, appearance, and privacy (Fang and Dong 1998). Lower scores indicated a more positive parent‐child relationship. The Cronbach's α in the current study was 0.860.

#### 2.2.5 Academic Procrastination Scale–Short Form

This instrument is designed to gauge academic procrastination through five items. Participants responded to these items using a 5‐point Likert‐type scale, ranging from 1 (total disagreement) to 5 (total agreement). Scores range from 5 to 25, with higher values indicating increased levels of academic procrastination (Yockey 2016). The Cronbach's α of the Academic Procrastination Scale in this study was 0.889.

### 2.3 Data Analysis

SPSS 26.0 was used for Harman's one‐factor test and descriptive statistics of statistical demographic characteristics in this study. Data analysis was conducted using partial least squares structural equation modeling (PLS‐SEM), a method employed for predictive analysis and theoretical development. This aligns with our objective to elucidate the underlying mechanisms between mobile phone addiction and academic procrastination based on SCT. Furthermore, PLS‐SEM is robust for estimating complex models involving multiple mediating and moderating effects, making it well‐suited for testing our proposed framework (Becker et al. 2023; Ghasemy et al. 2020). Specifically, the systematic procedure for applying the PLS‐SEM methodology consists of a two‐step process of assessing the quality of the measurement model and the predictive ability of the structural model. The software used to perform the analysis is SmartPLS 4.0.

## 3 Results

### 3.1 Common Method Bias Test

Non‐rotated exploratory factor analysis was performed on all indicators using the Harman one‐way test before processing data (Podsakoff et al. 2003). The results showed that 6 factors’ eigenvalues were larger than 1. The load of the first factor was 35.212%, which was < 40% of the covariance among all the items. It suggested that there was no serious common method bias in this study.

### 3.2 Sample Characteristics

According to **Table** **1**, participants were 19.7% (190) male and 80.3% (776) female. 25% of the participants were middle school students, and 75% were high school students. The remaining demographic characteristics are shown in **Table** **1**.

**TABLE 1:** Demographic characteristics of the sample (*N* = 966).

Category	Category description	Frequency	Percentage (%)
Gender	Male	190	19.7
	Female	776	80.3
Grade level	Grade 7 (Junior 1)	39	4
	Grade 8 (Junior 2)	149	15.4
	Grade 9 (Junior 3)	54	5.6
	Grade 10 (Senior 1)	349	36.1
	Grade 11 (Senior 2)	368	38.1
	Grade 12 (Senior 3)	7	0.7
Residence	Urban	366	37.9
	Rural	600	62.1
Number of siblings	0	341	35.3
	1	461	47.7
	2	130	13.5
	3 or more	34	3.5
Annual household income	≤100,000 RMB	728	75.4
	100,000–200,000 RMB	162	16.8
	200,000–500,000 RMB	50	5.2
	≥ 500,000 RMB	26	2.7
School type	Private	25	2.6
	Public	941	97.4

### 3.3 Measurement Model Assessment

In the measurement model, the Variance Inflation Factor (VIF) values of the variables were first used to quantify the severity of the covariance problem. The VIF values of the question items in this study fall between 1.457 and 4.032, which are less than the very strict empirical value of 5. This indicates that there is no high correlation between the apparent variables; that is, the multicollinearity is not significant. Meanwhile, Cronbach's α, Composite Reliability (CR), and Average Variance Extracted (AVE), as well as loading coefficients, were used to measure the validity of the measurement model. The Cronbach's α for each significant variable—Academic Procrastination (AP), Mobile Phone Addiction (MPA), Self‐Regulation (SR), Psychological Resilience (PR), and Parent‐Child Relationship (PCR) —is 0.889, 0.942, 0.915, 0.896, and 0.860, respectively, and the values are all greater than 0.7, which indicates that the reliability of the data is good. Meanwhile, the CR values were 0.919, 0.950, 0.927, 0.920, and 0.890, with values greater than 0.7, indicating that the attribution of individual question items to their respective variables is reasonable. In addition, the AVE values for convergent validity were 0.693, 0.634, 0.560, 0.660, and 0.505, which were all greater than 0.5, indicating that the data had good convergent validity. Further analyzing the discriminant validity of the data, the correlation coefficients of each variable were less than the open square root of the AVE of the respective variable, suggesting that the question items of each variable can be well differentiated. Meanwhile, the factor loading coefficients of each question item were greater than the 0.5 threshold and reached the significant level (all greater than 1.96). In addition, we also examined the Heterotrait‐Monotrait ratios, and **Table** **2** shows that all values are below the threshold of 0.90. Therefore, the results of the study indicate that there is no issue of discriminant validity in this study, and values below 0.9 indicate a more pronounced discriminant situation. Based on the above analysis of the results, the measurement model results passed the test, and further structural modeling tests can be conducted.

**TABLE 2:** Heterotrait‐Monotrait ratio.

	MPA	SR	PR	PCR	AP
MPA	0.796				
SR	−0.369	0.748			
PR	−0.406	0.610	0.812		
PCR	0.509	−0.500	−0.362	0.711	
AP	0.592	−0.551	−0.531	0.515	0.833

Abbreviations: MPA, Mobile Phone Addiction; SR, Self‐Regulation; PR, Psychological Resilience; PCR, Parent‐Child Relationship; AP, Academic Procrastination.

### 3.4 Structural Model Assessment

In Smart‐PLS 4.0, the coefficients of determination index (*R* ^2^), standardized root mean square residual (SRMR) and Normed Fit Index (NFI) are the main indicators for the validity test of the structural model. The larger the *R* ^2^ is, the stronger the explanatory strength of the endogenous latent variable is explained by the exogenous latent variable, and the better the validity of the structural model is. The *R* ^2^ of psychological resilience, academic procrastination, and self‐regulation are 0.408, 0.522, and 0.135, respectively, which are all higher than 0.1, indicating that the explanatory strength of the model is acceptable; meanwhile, the SRMR is 0.092, and the NFI is 0.823, which are all within the threshold of the model fit index. Overall, the model fit is good.

Next, this study further sampled 5000 times by the bootstrapping method and tested the initial model using a two‐tailed *t*‐test to obtain the correlation path coefficients, standard deviations, *T‐*values, and *p‐*values between the latent variables. Generally, test results with a *T*‐value greater than 1.96 or *a p‐*value less than 0.05 indicate that the model passes the significance test.

**FIGURE 2:**
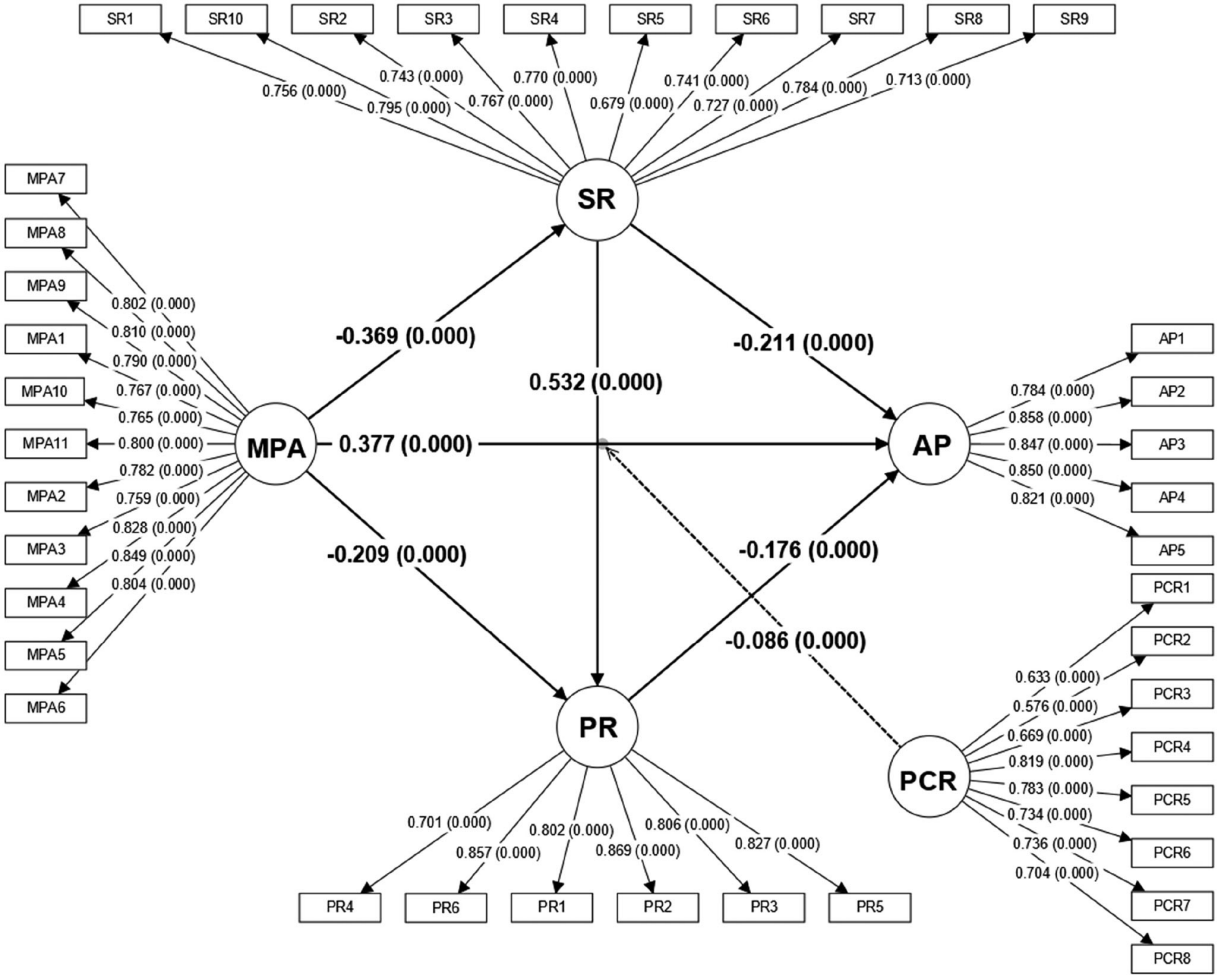
Results of the hypothetical research model. Abbreviations: MPA, Mobile Phone Addiction; SR, Self‐Regulation; PR, Psychological Resilience; PCR, Parent‐Child Relationship; AP, Academic Procrastination.

In **Figure** **2** and **Table** **3**, the path analysis revealed significant associations among variables. The results of the structural model analysis provided strong empirical support for the framework grounded in SCT. Mobile phone addiction had a significant positive impact on academic procrastination (*β* = 0.377, *p* < 0.001) confirmed H1, indicating that harmful behavioral habits exert a direct influence on academic performance.

**TABLE 3:** Outcomes of structural model.

	*β*	SD	*T*	*P*	LLCI	ULCI	Hypothesis	Result
**Direct effects**								
MPA→AP	0.377	0.032	11.937	< 0.001	0.315	0.438	H1	Supported
MPA→PR	−0.209	0.034	6.132	< 0.001	−0.277	−0.143		
MPA→SR	−0.369	0.032	11.585	< 0.001	−0.432	−0.306		
PR→AP	−0.176	0.039	4.467	< 0.001	−0.254	−0.098		
SR→AP	−0.211	0.037	5.641	< 0.001	−0.284	−0.138		
SR→PR	0.532	0.037	14.278	< 0.001	0.457	0.605		
**Indirect effects**								
MPA→SR→AP	0.078	0.015	5.327	0.001	0.050	0.110	H2	Supported
MPA→PR→AP	0.037	0.011	3.464	< 0.001	0.019	0.061	H3	Supported
MPA→SR→PR→AP	0.034	0.009	3.983	0	0.019	0.054	H4	Supported
**Moderating effect**								
PCR × MPA→AP	−0.086	0.023	3.806	< 0.001	−0.133	−0.043	H5	Supported

Abbreviations: MPA, Mobile phone addiction; SR, Self‐Regulation; PR, Psychological Resilience; PCR, Parent‐Child Relationship; AP, Academic Procrastination; LLCI, Lower limit of confidence interval; ULCI, Upper limit of confidence interval.

Three significant indirect impact paths from mobile phone addiction to academic procrastination were identified, and all of these mediation effects were partial mediation. Specifically, a significant indirect path was observed from mobile phone addiction to academic procrastination via self‐regulation (*β* = 0.078, *p* < 0.01), supporting H2; The indirect effect through psychological resilience was also significant (*β* = 0.037, *p* < 0.001), supporting H3. These findings reveal the mechanisms through which individual psychological resources influence problem behaviors. Thus, reduced psychological resilience functions as a vulnerability mechanism contributing to greater procrastination. Furthermore, mobile phone addiction and academic procrastination were connected through a serial path involving self‐regulation and psychological resilience (*β* = 0.034, *p* < 0.001) confirming H4, revealing a cascade effect whereby mobile phone addiction undermines self‐regulation capacity, thereby weakening resilience to stress, and ultimately leads to more severe procrastination behavior.

In addition, the significant negative interaction term between mobile phone addiction and the parent‐child relationship on academic procrastination (*β* = −0.086, *p* < 0.001) confirmed the moderating effect posited in H5, suggesting that a positive parent‐child relationship can buffer the negative impact of mobile phone addiction.

Overall, all hypotheses were supported by the data, and the relationships among variables were consistent with the SCT framework.

## 4 Discussion

This study constructed a dual‐mediation model linking mobile phone addiction to academic procrastination through self‐regulation and psychological resilience. Combined with the moderating role of parent‐child relationship, it provides a multi‐level explanatory framework for the understanding of adolescent procrastination behaviors. Findings indicated that mobile phone addiction not only directly leads to academic procrastination but also mediates this effect through diminished self‐regulation abilities and weakened psychological resilience; at the same time, the parent‐child relationship demonstrated a moderating function, wherein its strength was inversely correlated with the magnitude of the mobile phone addiction‐academic procrastination association. Overall, the findings align with the triadic reciprocity principle in SCT and extend previous research on the relationship between mobile phone addiction and academic procrastination, providing more effective guidance for reducing adolescents’ academic procrastination.

The current study verified that mobile phone addiction is a significant positive predictor of adolescent academic procrastination; that is, the more severe the adolescent's mobile phone addiction, the more likely the adolescent's academic procrastination behavior. These findings are consistent with Albursan et al. (2022) and Liu et al. (2022), indicating that the association between the two is a robust phenomenon. This underlying mechanism can be understood through the perspective of reinforcement and resource allocation. The instant gratification provided by smartphone applications (e.g., social media, games) is inherently more appealing than academic tasks requiring sustained effort and delayed gratification. Consequently, adolescents with mobile phone addiction are prone to allocate disproportionate time to their devices during non‐class hours (Yerdelen et al. 2016) and may resort to them as maladaptive coping strategies to escape academic pressures. This behavior directly reduces opportunities for academic engagement and triggers procrastination in their studies (Su et al. 2025; Zhou et al. 2024).

This study further confirmed the mediating role of self‐regulation and psychological resilience in the relationship between mobile phone addiction and academic procrastination among adolescents. Specifically, mobile phone addiction not only directly leads to academic procrastination but also indirectly increases its risk by weakening individuals’ self‐regulation abilities and psychological resilience. This indicated that impaired self‐regulation and psychological resilience serve as key cognitive mechanisms through which mobile phone addiction exacerbates academic procrastination. On the one hand, the close association between self‐regulation and mobile phone addiction can be explained through a neuro‐mechanism. Prior research indicated that problematic internet use is associated with gray matter reduction in brain regions such as the dorsolateral prefrontal cortex and anterior cingulate cortex, which constitute the neural basis for executive inhibitory control and reward processing (Solly et al. 2022). Excessive mobile phone use may damage these neural structures, thereby weakening the ability to plan academic tasks and resist distractions, ultimately manifesting as lower self‐regulation and higher academic procrastination (Li et al. 2020). On the other hand, mobile phone addiction deprives individuals of opportunities to develop psychological resilience. When facing stress or negative emotions, adolescents prone to mobile phone addiction habitually escape reality through phone use (e.g., endlessly scrolling through social media or playing games). While this avoidance coping provides temporary relief, it hinders the development of skills needed to actively regulate emotions, manage time, and form adaptive solutions under pressure (Sisto et al. 2019; Xu 2023). Generally, adolescents with stronger psychological resilience tend to exhibit greater willpower, employ proactive strategies to tackle academic challenges (Tang et al. 2021), and are less prone to academic procrastination. This indicated that mobile phone addiction is a risk factor for weakened psychological resilience, while low psychological resilience, in turn, increases the likelihood of academic procrastination.

Self‐regulation and psychological resilience were found to have a serial mediating effect between mobile phone addiction and academic procrastination in this study. This pathway reveals that the impact of mobile phone addiction on academic procrastination not only manifests as a direct time‐replacing effect but also involves an inherent mechanism where cognitive resources are consumed, thereby impeding the development of adaptive psychological skills. This pathway is supported by previous research; Stover et al. (2024) demonstrated that positive emotion regulation strategies and high‐level cognitive reappraisal skills can significantly enhance individuals’ psychological resilience. Excessive mobile phone use, however, continuously occupies individuals’ limited cognitive and attentional resources, which directly impairs their capacity for effective self‐regulation, such as impulse inhibition and goal maintenance (Wacks and Weinstein^2021^). The failure to develop adequate self‐regulatory abilities further deprives individuals of opportunities to accumulate resilience, exacerbating academic procrastination. This cascading effect provides a more nuanced explanation for why adolescent mobile phone addiction is difficult to eradicate and why its academic consequences are so severe.

It is worth noting that the mediation model we identified is partial rather than complete. This discrepancy suggests that other unmeasured factors may also explain the direct influence of mobile phone addiction on procrastination. For instance, Parmaksız's (2023) research on adolescents demonstrated that academic self‐efficacy also partially mediated the relationship between mobile phone use and academic procrastination, suggesting multiple psychological pathways are at play.

Parent‐child relationships were found to have a significant moderating effect, which substantiates the environmental component of SCT. This is in accordance with previous studies that have shown a supportive family environment, characterized by warmth and guidance, provided protective effects against adolescents’ problem behaviors (Mi et al. 2023). Adolescents in such environments are more likely to seek guidance and internalize adaptive values, reducing their reliance on phones for escapism and thereby weakening the pathway to procrastination (Brandhorst et al. 2021; Sun et al. 2022). Conversely, in conflictual or detached family contexts, adolescents may lack both the internal motivation and external support to regulate their phone use (Chung et al. 2019). This increases the likelihood that the mobile phone becomes a primary escape from academic pressures, thereby strengthening the association between addiction and procrastination (Yuan et al. 2022). Compared to individualistic cultural contexts, this buffering effect may be particularly significant in collectivist cultures, which highly emphasize family interdependence and academic achievement (Shek et al. 2019). Future cross‐cultural comparative studies may clarify whether these mechanisms are culturally specific or universal.

In summary, this study not only expands the application of SCT in the study of adolescent behavioral mechanisms but also provides empirical reference for psychological intervention and family education in middle school.

## 5 Implications and Limitations

Theoretically, this study complements and expands research on the associative mechanisms between mobile phone addiction and academic procrastination. The integrated model grounded in SCT provides empirical support for investigating academic procrastination among adolescents in the digital age and offers a reference for further multifactorial integrated research on adolescent problem behaviors. Methodologically, the integration of the PLS‐SEM method and the SCT framework captures complex interdependencies often overlooked in regression studies and provides a new perspective on the complex interactions among variables. From a practical standpoint, our findings may help guide prevention and intervention efforts for academic procrastination among adolescents. Research indicates that efforts to reduce academic procrastination may be more effective when shifting from single‐pronged approaches—such as solely targeting phone usage or actively cultivating adolescents’ intrinsic cognitive resources—to a dual‐pronged strategy. On one hand, cognitive‐behavioral techniques (e.g., emotions and time management) should be employed to strengthen adolescents’ self‐regulation abilities and psychological resilience. On the other hand, reinforcing the parent‐child relationship is crucial as a protective buffer within the environment. For schools, this implies the necessity of integrating adaptive coping and self‐regulation modules into the curriculum. For parents, the core intervention should not only focus on managing adolescents’ phone usage but also emphasize building emotional support for their academic and personal lives through open and warm communication. The coordinated implementation of these strategies may enhance adolescents’ academic performance and support their personal development.

Several limitations of the current study should be noted. First, the study adopts a cross‐sectional design, which only provides a prediction of the relationship between the variables. A subsequent longitudinal follow‐up study based on this study could reveal the causal paths between variables more precisely, thus clarifying the dynamic mechanisms of the factors in the time dimension. Second, although anonymity and attention‐check items were implemented to mitigate bias, the reliance on self‐report measures remains susceptible to social desirability biases. Future research could incorporate multi‐informant data (e.g., from parents or teachers) to enhance the validity of findings. Third, the convenience sampling method primarily covers adolescent populations in certain provinces of China, with a higher proportion of females in the sample. This may limit the generalizability of the research findings. Future studies should adopt multicenter random sampling strategies to incorporate more diverse populations representing different educational stages, regions, urban and rural areas, and balanced gender distributions. Thus, caution is needed when generalizing these findings to other populations.

## 6 Conclusion

This study used the PLS‐SEM method to reveal the complex associations among mobile phone addiction, academic procrastination, self‐regulation, psychological resilience, and the parent‐child relationship. Grounded in SCT, the integrated model demonstrates how personal resources and environmental contexts interact to shape adolescent behavior. The results illustrated serial mediation pathways through self‐regulation and psychological resilience, while also highlighting the buffering role of the parent‐child relationship. Collectively, these findings emphasize that while cultivating adolescents’ emotional regulation and self‐monitoring capacities, it is imperative to simultaneously foster supportive parent‐child communication. Implementing multi‐level interventions that target both individual skills and the family environment is crucial for mitigating mobile phone dependency and academic procrastination, thereby promoting healthier academic engagement.

## Author Contributions

**Yang Liu**: conceptualization, data curation, writing – original draft, and writing – review and editing. **Yan Lin**: conceptualization, data curation, writing – original draft, writing – review and editing. **Shaokun Zhao**: software, formal analysis, writing – review and editing. **Fan Wang**: writing – review and editing. **Qingying Yuan**: data curation. **Yongsheng Tong**: conceptualization, project administration, resources, and supervision.

## Funding

The present study was supported by the Beijing Research Ward Excellence Program (Grant Number: BRWEP2024W072130101).

## Data Availability

The de‐identified dataset and analysis code supporting this study are available on request to the corresponding author.
